# Analysis of the *In Vivo* Turnover of CD4+ T-Cell Subsets in Chronically SIV-Infected Sooty Mangabeys

**DOI:** 10.1371/journal.pone.0156352

**Published:** 2016-05-26

**Authors:** Alexandra M. Ortiz, Diane G. Carnathan, Joana Yu, Katherine M. Sheehan, Peter Kim, Arnold Reynaldi, Thomas H. Vanderford, Nichole R. Klatt, Jason M. Brenchley, Miles P. Davenport, Guido Silvestri

**Affiliations:** 1 Yerkes National Primate Research Center, and Emory Vaccine Center, Emory University, Atlanta, 30322, Georgia, United States of America; 2 Complex Systems in Biology Group, Centre for Vascular Research, University of New South Wales, Sydney, 2052, Australia; 3 Laboratory of Molecular Microbiology, National Institutes of Health, Bethesda, 20892, Maryland, United States of America; 4 Department of Pharmaceutics, University of Washington, Seattle, 98195, Washington, United States of America; University of Pittsburgh Center for Vaccine Research, UNITED STATES

## Abstract

Aberrant turnover of memory CD4+ T-cells is central to Acquired Immunodeficiency Syndrome (AIDS) progression. Understanding the relationship between the turnover of CD4+ subsets and immunological homeostasis during simian immunodeficiency virus (SIV) infection in natural hosts may provide insight into mechanisms of immune regulation that may serve as models for therapeutic intervention in Human Immunodeficiency Virus (HIV)-infected persons. Sooty mangabeys (SMs) have naturally evolved with SIV to avoid AIDS progression while maintaining healthy peripheral CD4+ T-cell counts and thus represent a model by which therapeutic interventions for AIDS progression might be elucidated. To assess the relationship between the turnover of CD4+ subsets and immunological homeostasis during SIV infection in non-progressive hosts, we treated 6 SIV-uninfected and 9 SIV-infected SMs with 2’-bromo-5’-deoxyuridine (BrdU) for 14 days and longitudinally assessed CD4+ T-cell subset turnover by polychromatic flow cytometry. We observed that, in SIV-infected SMs, turnover of CD4+ T-cell naïve and central, transitional, and effector memory subsets is comparable to that in uninfected animals. Comparable turnover of CD4+ T-cell subsets irrespective of SIV-infection status likely contributes to the lack of aberrant immune activation and disease progression observed after infection in non-progressive hosts.

## Introduction

Pathogenic human immunodeficiency virus (HIV) infection of humans and SIV infection of Asian macaques are associated with chronic immune activation and accelerated CD4+ T cell turnover [1–5]. CD4+ T-cells are comprised of naive (T_N_) and memory (i.e., antigen experienced) cells. Within the pool of memory CD4+ T-cells, central-memory T-cells (T_CM_) are a population of long-lived cells which can reside in secondary lymphoid tissues and are able to both self-regenerate and to differentiate into shorter-lived effector-memory T-cells (T_EM_) that home preferentially to tissues and mediate direct effector functions [6]. Cells expressing functional and phenotypic properties that are intermediate between T_CM_ and T_EM_ are defined as transitional-memory T cells or T_TM_. It was recently proposed that, during pathogenic HIV/SIV infections, high levels of direct virus infection of T_CM_ contribute to the progressive depletion of total CD4+ T-cells which is typically associated with progression to AIDS [7–10]. Non-pathogenic SIV infection of sooty mangabeys (SM), an African "natural" host species, is typically characterized by high virus replication, absence of chronic immune activation [11], and lower levels of CD4+ T_CM_ infection, with the latter finding having been attributed at least in part to lower expression of the SIV co-receptor CCR5 on SM CD4+ T_CM_ [9]. Consistent with the low immune activation of SIV-infected SMs is the observation of similar rates of “bulk” CD4+ and CD8+ T cell proliferation and death in SIV-uninfected and infected animals as measured by *in vivo* administration of the thymidine analogue BrdU and longitudinal analysis of its levels of incorporation and decay [12].

In this study, we used *in vivo* BrdU labeling to directly measure the rates of proliferation and death of CD4+ naive (T_N_), T_CM_, T_TTM_, and T_EM_, as well as CD4+CCR5+ memory T-cells in SIV-uninfected and infected SM to determine whether SIV infection of SMs is associated with a selectively increased turnover of specific CD4+ T cell subsets. In contrast to previously reported findings in pathogenically infected rhesus macaques, we observed that SIV infection in mangaeys was not associated with accelerated bulk or subset CD4+ T-cell proliferation or turnover. These results further support the hypothesis that a preserved CD4+ T-cell compartment contributes to natural host resistance to AIDS progression

## Materials and Methods

### Ethics Statement

The Institutional Animal Care and Use Committee of Emory University approved all of the experimental procedures (protocol numbers #254-2007Y and #YER-2000526-031014GA). The Committee complies with all applicable provisions of the Animal Welfare Act and other federal statutes and regulations relating to animals. Animals were housed at the Yerkes National Primate Research Center and maintained in accordance with guidelines set forth by the National Institutes of Health (NIH), under the supervision of the Association for the Assessment and Accreditation of Laboratory Animal Care (AAALAC)-accredited Division of Animal Resources and as recommended by the NIH Office of Animal Care and Use Nonhuman Primate Management Plan. Husbandry and care met the standards set forth by the Animal Welfare Act, Animal Welfare Regulations, as well as The Guide for the Care and Use of Laboratory Animals (8^th^ Edition). To minimize suffering during blood collection, animals were anesthetized with Ketamine (10 mg/kg IM) or Telazol (5 mg/kg IM). Successful anesthetization was monitored by response to stimuli. The physical conditions of the animals were monitored daily. No animal exhibited physical or behavioral evidence of BrdU toxicity or illness throughout the study period and thus, did not approach humane endpoint criteria as established by the Emory IACUC. Endpoint criteria included presentation of any of the following: (a) loss of 25% body weight from baseline weight when assigned to the protocol, (b) major organ failure or medical conditions unresponsive to treatment, (c) complete anorexia for 4 days or an inability to feed or drink sufficient nutrients to maintain body weight without assistance for 7 days, (d) distress vocalization unresponsive to treatment or intervention for 7 days, or (e) tumors arising from other than experimental means that grow in excess of 10% of body weight, impair movement, or ulcerate. To minimize undue stress, animals were provided enrichment such as foraging for grains, Kong^™^ toys and other enrichment deemed necessary after observations made by the Yerkes Behavioral Management Unit. Additionally, where possible, animals were housed with panels to allow some social contact for grooming and other social activities.

### Animals and BrdU Treatment

Six healthy, SIV-uninfected and nine naturally SIV_smm_-infected SMs were used in this study and included animals uniquely identified as: FBn, FBs, FCs, FEa1, FGn, FIa1, FJa1, FKa1, FKu, FLj, FSo, FSs, FWk, FWo, and FYs. All animals were negative for simian T-cell lymphotropic vIrus and either homozygous or heterozygous for the wt-*ccr5* allele [13]. Infected animals exhibited viral loads greater than 10,000 copies/ml and CD4+ T-cell counts greater than 500/μl and were naturally or experimentally infected for greater than four years. All animals exhibited comparable distributions of T-lymphocyte counts (data not shown). Animals were treated with 60 mg/kg i.v. of BrdU (Sigma-Aldrich) diluted in Hank’s buffered saline solution (Invitrogen) per weekday and 120 mg/kg orally of BrdU per weekend-day over 14 consecutive days. No evidence of BrdU toxicity was noted.

### Sample Processing and Immunophenotyping

Peripheral blood mononuclear cells (PBMCs) were stained for surface markers in whole blood and red blood cells were subsequently lysed with 1X BD FACS Lysing Solution (BD Biosciences Pharmingen). Cells were permeabilized with 1X Fixation/Permeabilization solution and washed with 1X BD Perm/Wash Buffer (BD Biosciences Pharmingen) prior to intracellular staining. Intracellular staining for Ki67 and BrdU included simultaneous treatment with 90 ug DNase I (Sigma-Aldrich) for 30min at 37°C. Polychromatic flow cytometry analysis was performed on an LSR II equipped with the FACs DiVA software (v6.1.3, BD) within 24 hours of sample collection. Mouse-derived monoclonal antibodies directed against the following antigens were used at titration-assessed volumes as follows: BrdU (clone 3D4, 20uL), CCR5 (3A9, 10uL), CD3 (SP34-2, 5uL), CD8 (SK1, 5uL), CD62L (SK11, 10uL), Ki67 (B56, 20uL) from BD Biosciences Pharmingen, CD4 (OKT4, 5uL) from BioLegend, CD28 (CD28.2, 10uL) from Beckman-Coulter, and CD95 (DX2, 10uL) from eBioscience. Rat-derived monoclonal antibody 3D12 (10uL) from BD was used to detect CCR7. Cell viability was determined using an amine-reactive fluorescent dye (Invitrogen). The data acquired was analyzed using FlowJo software (v9.4.4; TreeStar).

### Mathematical Modeling

The fraction of BrdU labeled cells over time was fitted to the previously described [3] three-parameter αρδ as follows:

$$L_{-1}(t)=\alpha \left(1-e^{-(d+p)(t-t_{0})}\right),\;t\leq 0,$$

$$L_{1}(t)=L_{-1}(0)e^{-(d-p)t},\;t\geq 0,$$

where *L*_*-1*_*(t)* is the fraction of labeled cells at time *t* during BrdU labeling, and L_1_(t) is the fraction of labeled cells at time *t* after BrdU administration has ended. We assume that BrdU labeling starts on day -14 (*t*_*0*_) and ends at day zero. The model considers parameter α, which represents the fraction of a cell population that would be labeled if BrdU were provided indefinitely, and ρ and δ, which represent the proliferation and death rates of the labeled populations, respectively. Average cell turnover ($\hat{\delta }$) was measured as $\hat{\delta }=\alpha \delta$, per day of the defined cell population [3].

### Statistical Analysis

Statistics were performed using Prism (v5.0c; GraphPad Software Inc.) or SAS 9.1. (SAS Institute Inc). The Mann-Whitney (one-way) test was performed to determine if BrdU incorporation or Ki67 expression in T-cell subsets were significantly different between infection groups. Averaged data are represented as arithmetic mean ± SEM. *p*-values less that 0.05 were considered significant.

## Results

**Fig 1:**
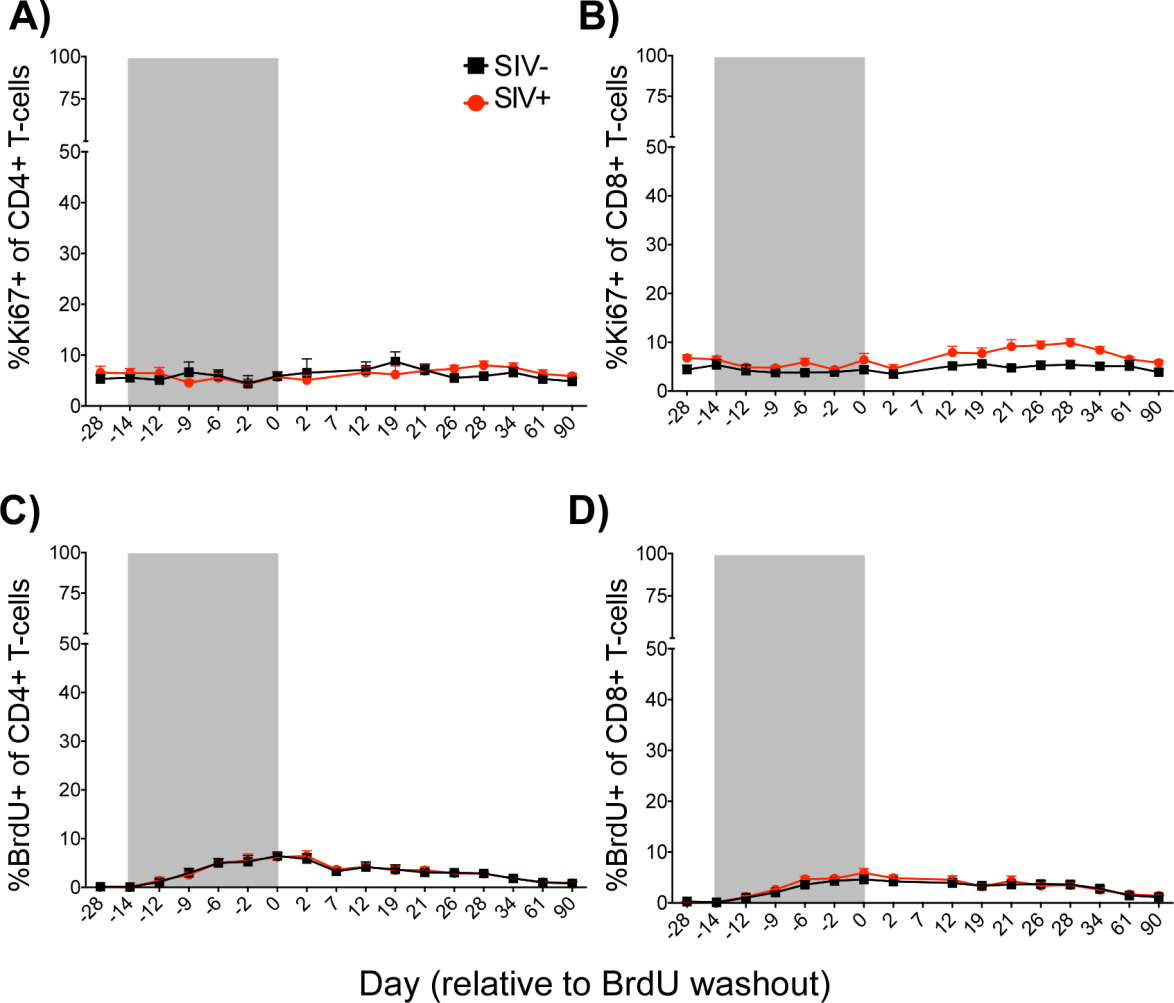
Ki67 and BrdU Expression Among Bulk Peripheral Blood Lymphocytes. The mean (±SEM) percent Ki67+ (A+B) and percent BrdU+ (C+D) of peripheral blood CD4+ (A+C) and CD8+ (B+D) T-cells were assessed longitudinally by flow cytometry in both uninfected (black squares) and infected (red circles) animals. *p* = NS between infected and uninfected controls at all timepoints for all populations (Mann-Whitney U). Shaded area represents BrdU administration period. Data points are shown only for animals above which 100 events were collected for the parent population.

We first examined the overall levels of expression of the proliferation marker Ki67 in "bulk" (i.e., total) peripheral blood CD4+ and CD8+ T-cells by flow cytometry as previously described [14]. Frequencies of Ki67+ T-cells remained stable over the experimental period, and did not show any significant difference between SIV-uninfected and infected animals (Fig 1A and 1B). We next measured frequencies of BrdU+ cells among total CD4+ and CD8+ T-cells during the infusion and thereafter and observed a rise and fall in frequencies of BrdU+ T-cells concurrent with the labeling and washout phases of this experiment (Fig 1C and 1D). We did not observe any significant difference in the kinetics of BrdU incorporation or loss of BrdU+ T-cells in total CD4+ or CD8+ T-cells, consistent with previous findings [12], and in keeping with the paradigm that non-pathogenic SIV infection of SMs is not associated with increased overall levels of T cell activation.

**Fig 2:**
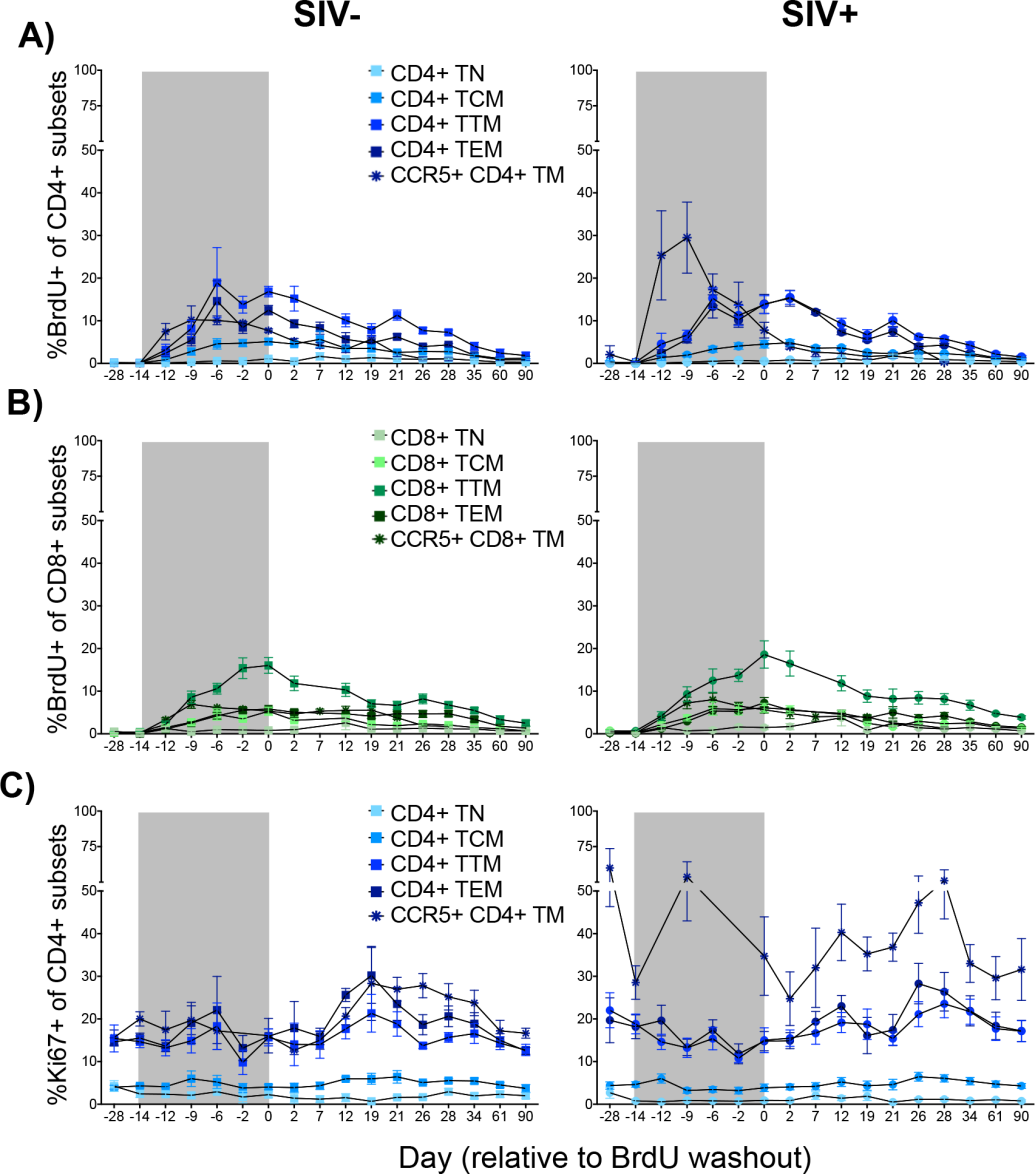
BrdU and Ki67 Expression By CD4+ and CD8+ T-cell Subsets. A-B. (±SEM) percent BrdU expression was assessed longitudinally by flow cytometry among CD4+ (A) and CD8+ (B) T_N_, T_CM_, T_TM_, T_EM_, and CCR5+ TM in uninfected (left) and SIV-infected (right) animals. C. Mean (±SEM) longitudinal Ki67 expression of CD4+ T_N_, T_CM_, T_TM_, T_EM_, and CCR5+ TM in uninfected (left) and SIV-infected (right) animals. *p* = NS between infected and uninfected controls at all timepoints for all populations (Mann-Whitney U). Shaded area represents BrdU administration period. Data points are shown only for animals above which 100 events were collected for the parent population.

To determine whether SIV infection of SMs is associated with a selectively increased turnover of certain T cell subsets, we next examined, in the same animals, the kinetics of BrdU incorporation and decay in T_N_, T_CM_, T_TM_, and T_EM_ subsets of CD4+ and CD8+ T-cells. Specifically, we hypothesized that a selectively increased turnover of CD4+ T_EM_ may be present during chronic SIV infection of SMs as a consequence of the fact that SIV infects these cells at higher frequency than other CD4+ T cell subsets [9]. In this study, T_N_ were defined as CD28+CD95-, T_CM_ were defined as CD95+CD62L+CCR7+, T_EM_ were defined as CD95+CD62L-CCR7-, and T_TM_ were defined as either CD95+CD62L+CCR7- or CD95+CD62L-CCR7+. As shown in Fig 2A, we observed no significant differences between SIV-uninfected (left) and infected (right) SMs with respect to the frequencies of BrdU+ CD4+ T_N_, T_CM_, T_TM_, or T_EM_ during the incorporation period though we did note a non-significant trend towards increased turnover of CD4+ T_EM_ in SIV-infected animals. Similarly, no significant differences were observed in the BrdU labeling or delabeling kinetics for any of the CD8+ T-cell subsets (T_N_, T_CM_, T_TM_, and T_EM_) between SIV-uninfected and infected SMs (Fig 2B).

Since primary SIV_smm_ isolates are predominantly CCR5-tropic and CD4+ memory T-cells (T_M_) represent the main cellular targets for the virus, we next investigated whether SIV-infected SMs show signs of increased turnover of circulating memory CD4+ T-cells expressing the SIV coreceptor CCR5. Of note, although the majority of CD4+CCR5+ T-cells in SMs are contained within the T_EM_ population, we included all CD4+CCR5+ memory T-cells in our analysis as CD4+CCR5+ T_CM_ are also potentially susceptible to SIV infection and, therefore, to the impact of the virus on the cell *in vivo* lifespan [9]. We observed that, similar to CD4+ T_EM_, CD4+CCR5+ T_M_ showed a non-significant trend towards higher frequencies of BrdU+ cells during the incorporation period in SIV-infected SMs as compared to uninfected animals (Fig 2A). No differences between SIV-infected and uninfected SMs were observed with respect to the level of BrdU incorporation and decay in CD8+CCR5+ memory T-cells (Fig 2B).

Longitudinal BrdU labeling is a surrogate measure of cellular turnover, a net reflection of proliferation and death. Although we did not observe any significant differences by infection status with regards to CD4+ T-cell subset BrdU labeling, we further investigated if differences in subset proliferation were evident. As measured by Ki67 expression, we did not observe any significant differences in CD4+ TN, TCM, TTM, TEM or CCR5+ TM proliferation between uninfected and SIV-infected animals although a non-significant trend for increased proliferation of CD4+ CCR5+ TM from SIV-infected animals was observed (Fig 2C). To more rigorously assess cellular turnover in populations of interest, we utilized three-parameter αρδ model [3]. We fit this model to BrdU expression values by bulk CD4+ T-cells, CD4+ TEM, CD4+ TCM and CD4+ CCR5+ TM subsets in our animals (Table 1) and calculated average turnover as previously described [3]. By this method, we did not observe any significant differences in the average turnover rate between similar subsets from SIV-infected and uninfected animals. Collectively, these data confirm that SIV infection of SMs is not associated with an increased turnover of "bulk" CD4+ or CD8+ T-cells, and expand upon these results by showing that SIV-infected animals do not exhibit a significantly increased cellular turnover in any of the studied T cell subpopulations (i.e., T_N_, T_CM_, T_TM_, T_EM_ or CCR5+ T_M_).

**Table 1:** a, p, and d values for CD4+ T-cell subsets.

**Table 1a: Labeling (a)**					
Animal	SIV Status	Bulk CD4	CD4 TCM	CD4 TEM	CCR5+ CD4+ TM
FBn	+	0.0389	0.0241	0.2047	0.1295
FBs	+	0.0476	0.0459	0.086	0.3024
FEa1	-	0.1387	0.0846	0.1	0.1541
FGn	+	0.0957	0.0559	0.133	0.1206
FKu	-	0.0844	0.0473	0.1243	0.1374
FLj	+	0.1581	0.0408	0.3648	0.2192
FSo	-	0.1423	0.1044	0.1649	0.1109
FWk	+	0.0506	0.0584	0.2099	0.2058
FYs	+	0.0567	0.1196	0.2863	0.4884
FCs	+	0.1565	0.1018	0.1599	0.03621
FSs	+	0.1887	0.1471	0.4953	0.1198
Fwo	+	0.1311	0.0328	0.2614	0.6519
FIa1	-	0.1817	0.06919	0.3389	0.09182
FJa1	-	0.08627	0.09482	0.1778	0.3008
FKa1	-	0.1535	0.1095	0.3241	0.03935
**Table 1b: Proliferation (p)**					
Animal	SIV Status	Bulk CD4	CD4 TCM	CD4 TEM	CCR5+ CD4+ TM
FBn	+	0.0973	0.0631	0.0039	0.1585
FBs	+	0.1	0.1584	0.0916	0
FEa1	-	0.0038	0.0339	0.0667	0.0515
FGn	+	0.113	0.1696	0.0705	0.2374
FKu	-	0.1443	0.1999	0.1575	0.0784
FLj	+	0	0.0518	0	0.4487
FSo	-	0	0.0048	0	0.3382
FWk	+	0.0571	0.0361	0.0222	4.356
FYs	+	0.0718	0.0156	0.0148	3.6092
FCs	+	7.556E-12	1.759E-12	0.02165	0.3202
FSs	+	1.28E-16	8.753E-16	6.744E-14	0.09404
Fwo	+	5.349E-13	0.03261	7.715E-13	0.4076
FIa1	-	0.03842	0.05173	0.06949	1.794E-13
FJa1	-	0.03094	0.06135	0.001382	0
FKa1	-	1.57E-16	2.689E-14	1.73E-16	0.08741
**Table 1c: Death (d)**					
Animal	SIV Status	Bulk CD4	CD4 TCM	CD4 TEM	CCR5+ CD4+ TM
FBn	+	0.1131	0.0695	0.0465	0.2608
FBs	+	0.1164	0.1742	0.1187	0.1473
FEa1	-	0.0241	0.0544	0.0967	0.1025
FGn	+	0.1379	0.184	0.0999	0.2775
FKu	-	0.1662	0.2188	0.1882	0.116
FLj	+	0.0383	0.0729	0.045	0.564
FSo	-	0.0277	0.024	0.0489	0.3807
FWk	+	0.098	0.0589	0.0835	4.453
FYs	+	0.1013	0.0511	0.0757	3.7423
FCs	+	0.03152	0.01852	0.05351	0.3581
FSs	+	0.0352	0.03564	0.05011	0.1745
Fwo	+	0.0316	0.04846	0.04372	0.5432
FIa1	-	0.03737	0.05496	0.05483	0.1336
FJa1	-	0.07716	0.08796	0.1223	0.06242
FKa1	-	0.03969	0.03559	0.03874	0.1016

## Discussion

Natural hosts have co-evolved with SIV resulting in a non-pathogenic infection despite high viremia. The key factors contributing to the benign nature of these infections are the absence of chronic immune activation and a different pattern of SIV cellular targeting *in vivo*, which preferentially preserves CD4+ T_CM_ and stem-cell memory cells or T_SCM_ [7–9, 15]. Based on the differential pattern of *in vivo* infected CD4+ T cell subsets in SIV-infected SMs vs. SIV-infected rhesus macaques (in which the infection is pathogenic), we had hypothesized that CD4+ T_EM_ and/or CD4+CCR5+ T_M_ of SMs may undergo faster turnover upon SIV infection. Our current set of data did not support this hypothesis per se, as the kinetics of BrdU incorporation and decay showed only a non-significant trend towards increased turnover for both cell subsets in SIV-infected SMs. We believe that two points should be made while discussing this experimental result. The first is that, due to the logistical and budgetary challenges of conducting this type of experiments in non-human primates, the current study had a limited statistical power to detect differences as significant between the two groups of animals. As such it is possible that the observed, non-significant increase in the turnover of CD4+ T_EM_ and CD4+CCR5+ T_M_ in SIV-infected SMs could have become significant in a study involving a larger number of animals. The second point is that, even within the subset of CD4+ T_EM_ that are preferentially infected in SMs, the fraction of SIV-DNA-positive cells is relatively low (i.e., consistently <1%, [9]), and therefore it is likely that the impact on the turnover of this cell population by virus-mediated cell death is simply too numerically small to be detected as a change in the kinetics of BrdU labeling and that de-labeling does not distinguish between virus-infected and uninfected cells. Importantly, the observation of similar rates of CD4+ T_CM_ proliferation and death in SIV-uninfected and infected SMs provides further support to the hypothesis that a preserved CD4+ T_CM_ compartment is central to the ability of SIV-infected SMs to avoid AIDS [8]. This result is also in stark contrast with the observation that CD4+ T_CM_ undergo accelerated turnover and ultimately lose their homeostasis in progressively SIV-infected rhesus macaques [10]. Understanding the molecular mechanisms responsible for the preservation of CD4+ T_CM_ and T_SCM_ homeostasis in SIV-infected SMs may help in the design of interventions aimed at preserving these important immune cell subsets and thus preventing disease progression in HIV-infected individuals.

## Supporting Information

S1 Table Data Underlying Primary Figures as Derived from Polychromatic Flow Cytometry Analysis of Peripheral Blood Lymphocytes. (XLSX)

## References

[1] Rosenzweig M, DeMaria MA, Harper DM, Friedrich S, Jain RK, Johnson RP. Increased rates of CD4(+) and CD8(+) T lymphocyte turnover in simian immunodeficiency virus-infected macaques. Proc Natl Acad Sci U S A. 1998;95(11):6388–93. 9600975 10.1073/pnas.95.11.6388 PMC27734

[2] Sachsenberg N, Perelson AS, Yerly S, Schockmel GA, Leduc D, Hirschel B, et al Turnover of CD4+ and CD8+ T lymphocytes in HIV-1 infection as measured by Ki-67 antigen. The Journal of experimental medicine. 1998;187(8):1295–303. 9547340 10.1084/jem.187.8.1295 PMC2212238

[3] De Boer RJ, Mohri H, Ho DD, Perelson AS. Turnover rates of B cells, T cells, and NK cells in simian immunodeficiency virus-infected and uninfected rhesus macaques. Journal of immunology (Baltimore, Md: 1950). 2003;170(5):2479–87. 10.4049/jimmunol.170.5.2479 12594273

[4] Hellerstein MK, Hoh RA, Hanley MB, Cesar D, Lee D, Neese RA, et al Subpopulations of long-lived and short-lived T cells in advanced HIV-1 infection. The Journal of clinical investigation. 2003;112(6):956–66. 10.1172/JCI17533 12975480 PMC193663

[5] Kovacs JA, Lempicki RA, Sidorov IA, Adelsberger JW, Herpin B, Metcalf JA, et al Identification of dynamically distinct subpopulations of T lymphocytes that are differentially affected by HIV. The Journal of experimental medicine. 2001;194(12):1731–41. 11748275 10.1084/jem.194.12.1731 PMC2193579

[6] Sallusto F, Lenig D, Förster R, Lipp M, Lanzavecchia A. Two subsets of memory T lymphocytes with distinct homing potentials and effector functions. Nature. 1999;401(6754):708–12. 10.1038/44385 10537110

[7] Brenchley JM, Vinton C, Tabb B, Hao XP, Connick E, Paiardini M, et al Differential infection patterns of CD4+ T cells and lymphoid tissue viral burden distinguish progressive and nonprogressive lentiviral infections. Blood. 2012;120(20):4172–81. 10.1182/blood-2012-06-437608 22990012 PMC3501715

[8] Chahroudi A, Bosinger SE, Vanderford TH, Paiardini M, Silvestri G. Natural SIV hosts: showing AIDS the door. Science. 2012;335(6073):1188–93. 10.1126/science.1217550 22403383 PMC3822437

[9] Paiardini M, Cervasi B, Reyes-Aviles E, Micci L, Ortiz AM, Chahroudi A, et al Low levels of SIV infection in sooty mangabey central memory CD(4)(+) T cells are associated with limited CCR5 expression. Nat Med. 2011;17(7):830–6. 10.1038/nm.2395 21706028 PMC3253129

[10] Okoye A, Meier-Schellersheim M, Brenchley JM, Hagen SI, Walker JM, Rohankhedkar M, et al Progressive CD4+ central memory T cell decline results in CD4+ effector memory insufficiency and overt disease in chronic SIV infection. J Exp Med. 2007;204(9):2171–85. 10.1084/jem.20070567 17724130 PMC2118701

[11] Silvestri G, Sodora DL, Koup RA, Paiardini M, O'Neil SP, McClure HM, et al Nonpathogenic SIV infection of sooty mangabeys is characterized by limited bystander immunopathology despite chronic high-level viremia. Immunity. 2003;18(3):441–52. . 12648460 10.1016/s1074-7613(03)00060-8

[12] Kaur A, Di Mascio M, Barabasz A, Rosenzweig M, McClure HM, Perelson AS, et al Dynamics of T- and B-lymphocyte turnover in a natural host of simian immunodeficiency virus. Journal of Virology. 2008;82(3):1084–93. 10.1128/JVI.02197-07 18032490 PMC2224458

[13] Riddick NE, Hermann EA, Loftin LM, Elliott ST, Wey WC, Cervasi B, et al A novel CCR5 mutation common in sooty mangabeys reveals SIVsmm infection of CCR5-null natural hosts and efficient alternative coreceptor use in vivo. PLoS Pathogens. 2010;6(8). 10.1371/journal.ppat.1001064 PMC2928783 20865163

[14] Klatt NR, Harris LD, Vinton CL, Sung H, Briant JA, Tabb B, et al Compromised gastrointestinal integrity in pigtail macaques is associated with increased microbial translocation, immune activation, and IL-17 production in the absence of SIV infection. Mucosal immunology. 2010;3(4):387–98. 10.1038/mi.2010.14 20357762 PMC2891168

[15] Cartwright EK, McGary CS, Cervasi B, Micci L, Lawson B, Elliott ST, et al Divergent CD4+ T memory stem cell dynamics in pathogenic and nonpathogenic simian immunodeficiency virus infections. Journal of immunology. 2014;192(10):4666–73. 10.4049/jimmunol.1303193 24729621 PMC4011949

